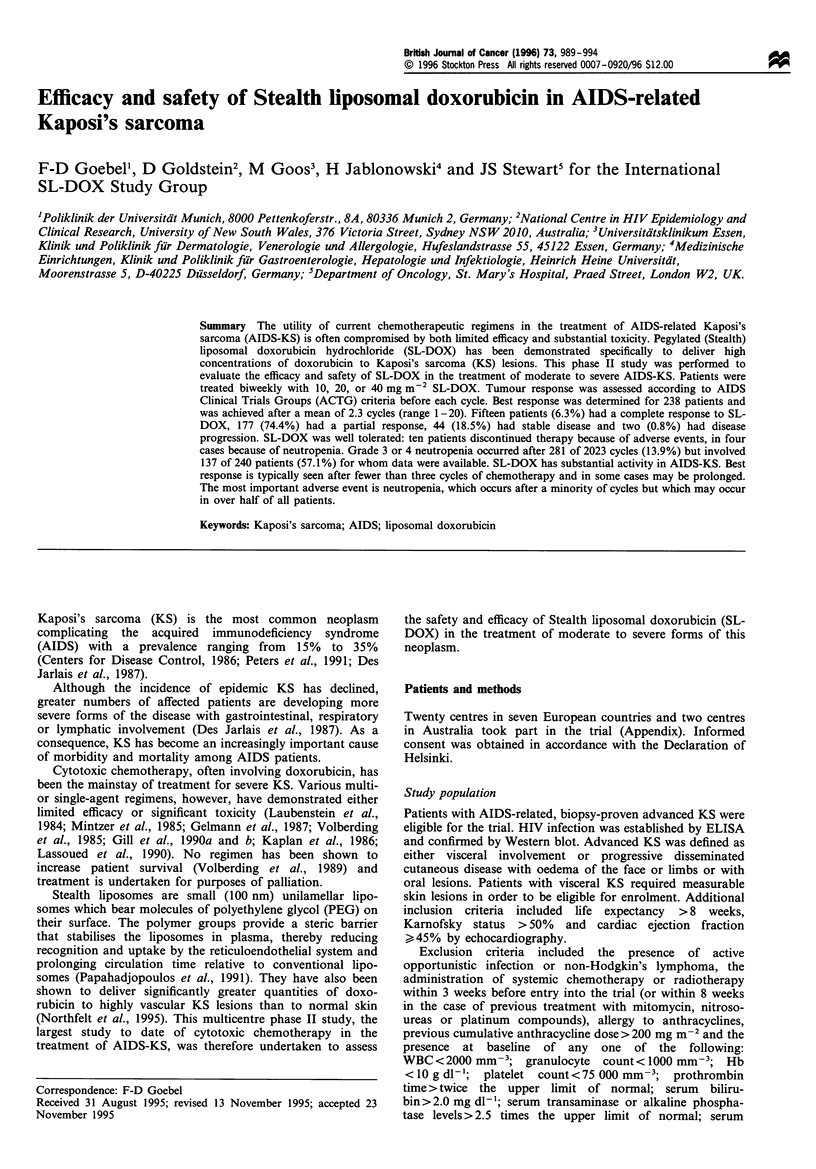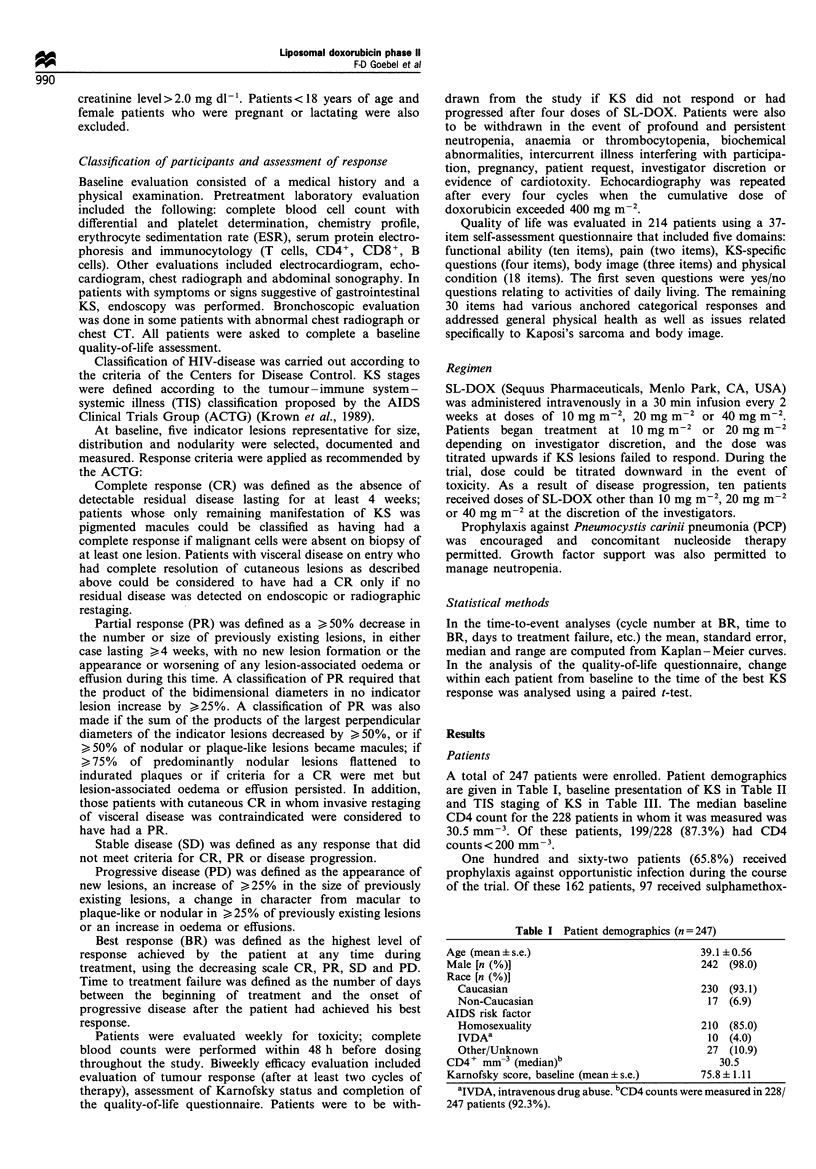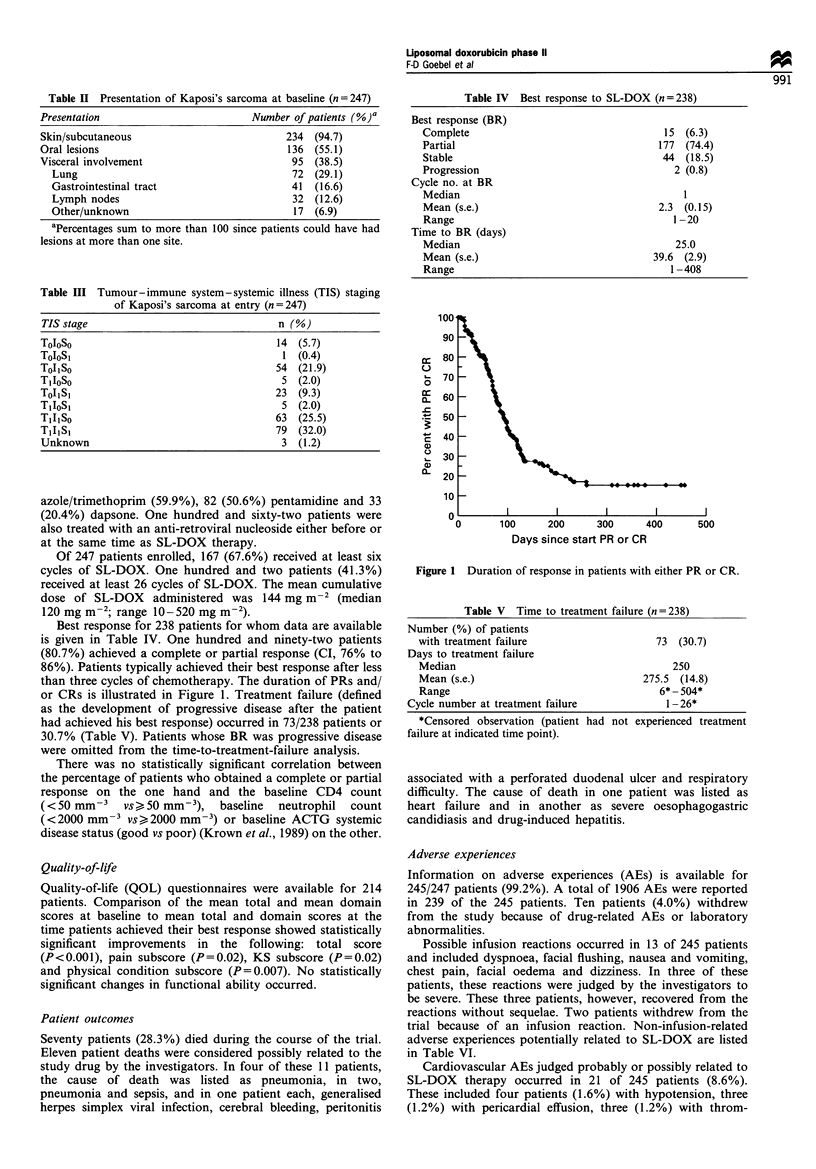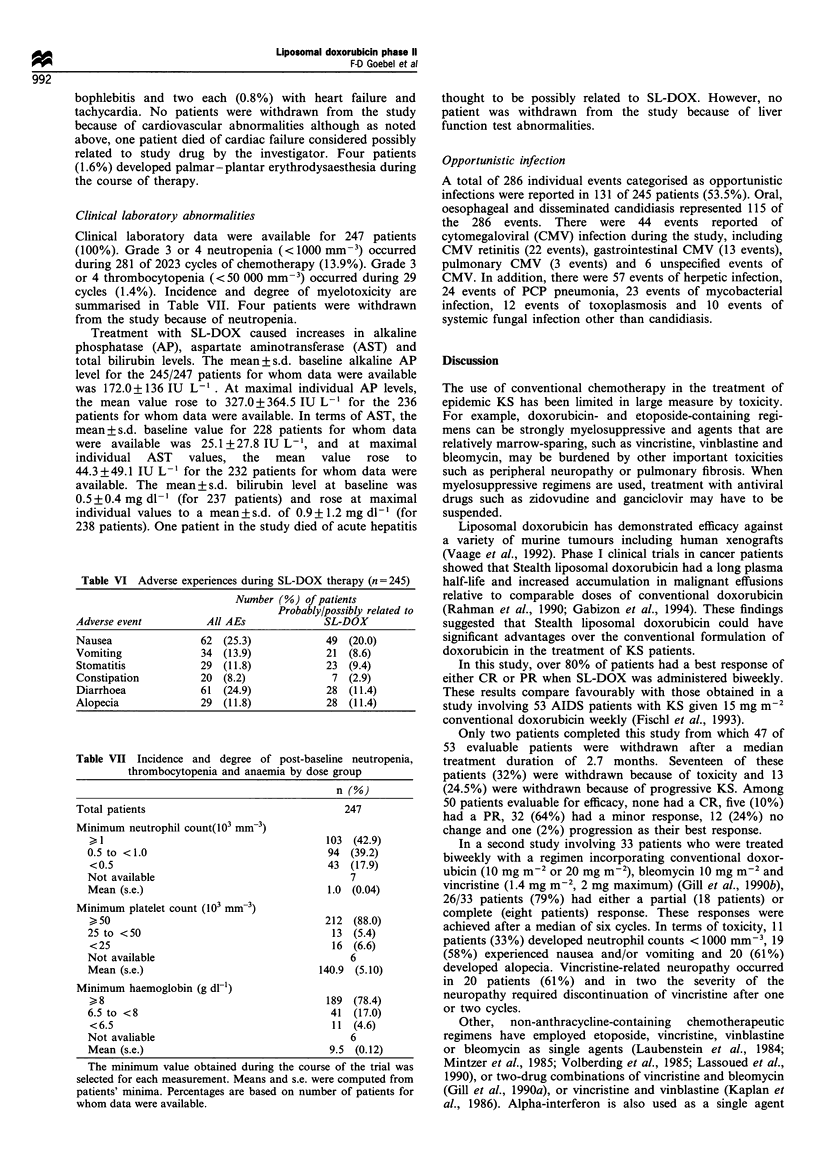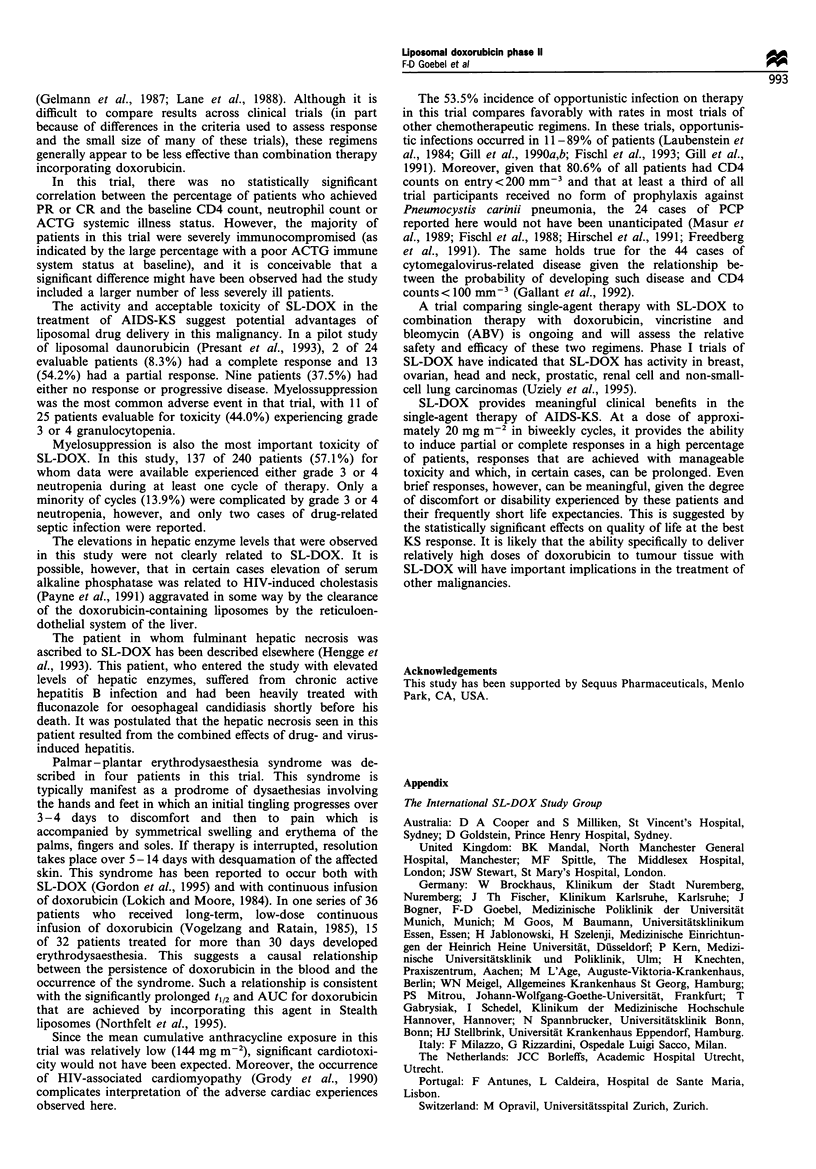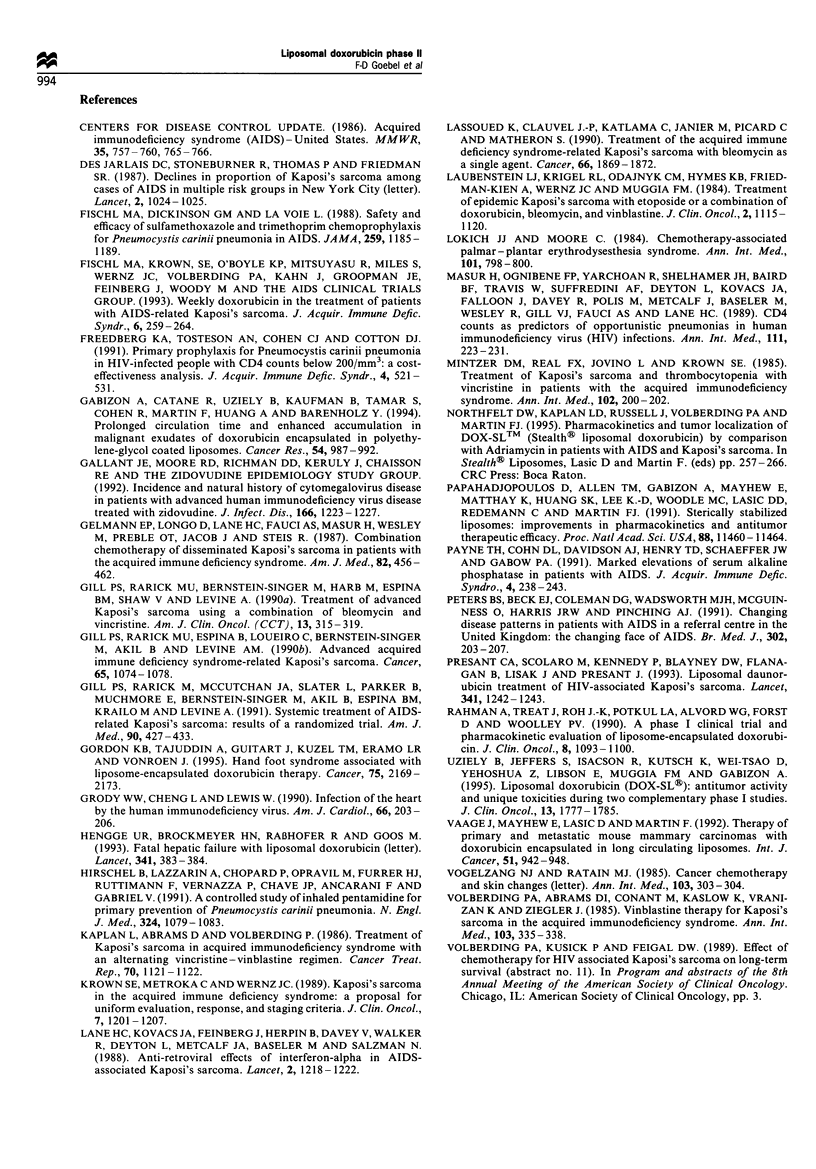# Efficacy and safety of Stealth liposomal doxorubicin in AIDS-related Kaposi's sarcoma. The International SL-DOX Study Group.

**DOI:** 10.1038/bjc.1996.193

**Published:** 1996-04

**Authors:** F. D. Goebel, D. Goldstein, M. Goos, H. Jablonowski, J. S. Stewart

**Affiliations:** Poliklinik der Universität Munich, Germany.

## Abstract

The utility of current chemotherapeutic regimens in the treatment of AIDS-related Kaposi's sarcoma (AIDS-KS) is often compromised by both limited efficacy and substantial toxicity. Pegylated (Stealth) liposomal doxorubicin hydrochloride (SL-DOX) has been demonstrated specifically to deliver high concentrations of doxorubicin to Kaposi's sarcoma (KS) lesions. This phase II study was performed to evaluate the efficacy and safety of SL-DOX in the treatment of moderate to severe AIDS-KS. Patients were treated biweekly with 10, 20, or 40 mg m-2 SL-DOX. Tumour response was assessed according to AIDS Clinical Trials Groups (ACTG) criteria before each cycle. Best response was determined for 238 patients and was achieved after a mean of 2.3 cycles (range 1-20). Fifteen patients (6.3%) had a complete response to SL-DOX, 177 (74.4%) had a partial response, 44 (18.5%) had stable disease and two (0.8%) had disease progression. SL-DOX was well tolerated: ten patients discontinued therapy because of adverse events, in four cases because of neutropenia. Grade 3 or 4 neutropenia occurred after 281 of 2023 cycles (13.9%) but involved 137 of 240 patients (57.1%) for whom data were available. SL-DOX has substantial activity in AIDS-KS. Best response is typically seen after fewer than three cycles of chemotherapy and in some cases may be prolonged. The most important adverse event is neutropenia, which occurs after a minority of cycles but which may occur in over half of all patients.


British Journal of Cancer (1996) 73, 989-994
© 1996 Stockton Press All rights reserved 0007-0920/96 $12.00

### Efficacy and safety of Stealth liposomal doxorubicin in AIDS-related Kaposi's sarcoma

F-D Goebel[1], D Goldstein[2], M Goos[3], H Jablonowski[4] and JS Stewart[5] for the International SL-DOX Study Group

[1]*Poliklinik der Universität Munich, 8000 Pettenkoferstr., 8A, 80336 Munich 2, Germany;* [2]*National Centre in HIV Epidemiology and Clinical Research, University of New South Wales, 376 Victoria Street, Sydney NSW 2010, Australia;* [3]*Universitätsklinikum Essen, Klinik und Poliklinik für Dermatologie, Venerologie und Allergologie, Hufeslandstrasse 55, 45122 Essen, Germany;* [4]*Medizinische Einrichtungen, Klinik und Poliklinik für Gastroenterologie, Hepatologie und Infektiologie, Heinrich Heine Universität, Moorenstrasse 5, D-40225 Düsseldorf, Germany;* [5]*Department of Oncology, St. Mary's Hospital, Praed Street, London W2, UK.*

**Summary** The utility of current chemotherapeutic regimens in the treatment of AIDS-related Kaposi's sarcoma (AIDS-KS) is often compromised by both limited efficacy and substantial toxicity. Pegylated (Stealth) liposomal doxorubicin hydrochloride (SL-DOX) has been demonstrated specifically to deliver high concentrations of doxorubicin to Kaposi's sarcoma (KS) lesions. This phase II study was performed to evaluate the efficacy and safety of SL-DOX in the treatment of moderate to severe AIDS-KS. Patients were treated biweekly with 10, 20, or 40 mg $m^{-2}$ SL-DOX. Tumour response was assessed according to AIDS Clinical Trials Groups (ACTG) criteria before each cycle. Best response was determined for 238 patients and was achieved after a mean of 2.3 cycles (range 1-20). Fifteen patients (6.3%) had a complete response to SL-DOX, 177 (74.4%) had a partial response, 44 (18.5%) had stable disease and two (0.8%) had disease progression. SL-DOX was well tolerated: ten patients discontinued therapy because of adverse events, in four cases because of neutropenia. Grade 3 or 4 neutropenia occurred after 281 of 2023 cycles (13.9%) but involved 137 of 240 patients (57.1%) for whom data were available. SL-DOX has substantial activity in AIDS-KS. Best response is typically seen after fewer than three cycles of chemotherapy and in some cases may be prolonged. The most important adverse event is neutropenia, which occurs after a minority of cycles but which may occur in over half of all patients.

**Keywords:** Kaposi's sarcoma; AIDS; liposomal doxorubicin

Kaposi's sarcoma (KS) is the most common neoplasm complicating the acquired immunodeficiency syndrome (AIDS) with a prevalence ranging from 15% to 35% (Centers for Disease Control, 1986; Peters *et al.*, 1991; Des Jarlais *et al.*, 1987).

Although the incidence of epidemic KS has declined, greater numbers of affected patients are developing more severe forms of the disease with gastrointestinal, respiratory or lymphatic involvement (Des Jarlais *et al.*, 1987). As a consequence, KS has become an increasingly important cause of morbidity and mortality among AIDS patients.

Cytotoxic chemotherapy, often involving doxorubicin, has been the mainstay of treatment for severe KS. Various multi- or single-agent regimens, however, have demonstrated either limited efficacy or significant toxicity (Laubenstein *et al.*, 1984; Mintzer *et al.*, 1985; Gelmann *et al.*, 1987; Volberding *et al.*, 1985; Gill *et al.*, 1990*a* and *b*; Kaplan *et al.*, 1986; Lassoued *et al.*, 1990). No regimen has been shown to increase patient survival (Volberding *et al.*, 1989) and treatment is undertaken for purposes of palliation.

Stealth liposomes are small (100 nm) unilamellar liposomes which bear molecules of polyethylene glycol (PEG) on their surface. The polymer groups provide a steric barrier that stabilises the liposomes in plasma, thereby reducing recognition and uptake by the reticuloendothelial system and prolonging circulation time relative to conventional liposomes (Papahadjopoulos *et al.*, 1991). They have also been shown to deliver significantly greater quantities of doxorubicin to highly vascular KS lesions than to normal skin (Northfelt *et al.*, 1995). This multicentre phase II study, the largest study to date of cytotoxic chemotherapy in the treatment of AIDS-KS, was therefore undertaken to assess the safety and efficacy of Stealth liposomal doxorubicin (SL-DOX) in the treatment of moderate to severe forms of this neoplasm.

Correspondence: F-D Goebel
Received 31 August 1995; revised 13 November 1995; accepted 23 November 1995

### Patients and methods

Twenty centres in seven European countries and two centres in Australia took part in the trial (Appendix). Informed consent was obtained in accordance with the Declaration of Helsinki.

### *Study population*

Patients with AIDS-related, biopsy-proven advanced KS were eligible for the trial. HIV infection was established by ELISA and confirmed by Western blot. Advanced KS was defined as either visceral involvement or progressive disseminated cutaneous disease with oedema of the face or limbs or with oral lesions. Patients with visceral KS required measurable skin lesions in order to be eligible for enrolment. Additional inclusion criteria included life expectancy >8 weeks, Karnofsky status >50% and cardiac ejection fraction ⩾45% by echocardiography.

Exclusion criteria included the presence of active opportunistic infection or non-Hodgkin's lymphoma, the administration of systemic chemotherapy or radiotherapy within 3 weeks before entry into the trial (or within 8 weeks in the case of previous treatment with mitomycin, nitrosoureas or platinum compounds), allergy to anthracyclines, previous cumulative anthracycline dose > 200 mg $m^{-2}$ and the presence at baseline of any one of the following: WBC < 2000 $mm^{-3}$; granulocyte count < 1000 $mm^{-3}$; Hb < 10 g $dl^{-1}$; platelet count < 75 000 $mm^{-3}$; prothrombin time > twice the upper limit of normal; serum bilirubin > 2.0 mg $dl^{-1}$; serum transaminase or alkaline phosphatase levels > 2.5 times the upper limit of normal; serum creatinine level > 2.0 mg $dl^{-1}$. Patients < 18 years of age and female patients who were pregnant or lactating were also excluded.

### *Classification of participants and assessment of response*

Baseline evaluation consisted of a medical history and a physical examination. Pretreatment laboratory evaluation included the following: complete blood cell count with differential and platelet determination, chemistry profile, erythrocyte sedimentation rate (ESR), serum protein electrophoresis and immunocytology (T cells, $CD4^+$, $CD8^+$, B cells). Other evaluations included electrocardiogram, echocardiogram, chest radiograph and abdominal sonography. In patients with symptoms or signs suggestive of gastrointestinal KS, endoscopy was performed. Bronchoscopic evaluation was done in some patients with abnormal chest radiograph or chest CT. All patients were asked to complete a baseline quality-of-life assessment.

Classification of HIV-disease was carried out according to the criteria of the Centers for Disease Control. KS stages were defined according to the tumour–immune system–systemic illness (TIS) classification proposed by the AIDS Clinical Trials Group (ACTG) (Krown *et al.*, 1989).

At baseline, five indicator lesions representative for size, distribution and nodularity were selected, documented and measured. Response criteria were applied as recommended by the ACTG:

Complete response (CR) was defined as the absence of detectable residual disease lasting for at least 4 weeks; patients whose only remaining manifestation of KS was pigmented macules could be classified as having had a complete response if malignant cells were absent on biopsy of at least one lesion. Patients with visceral disease on entry who had complete resolution of cutaneous lesions as described above could be considered to have had a CR only if no residual disease was detected on endoscopic or radiographic restaging.

Partial response (PR) was defined as a ⩾50% decrease in the number or size of previously existing lesions, in either case lasting ⩾4 weeks, with no new lesion formation or the appearance or worsening of any lesion-associated oedema or effusion during this time. A classification of PR required that the product of the bidimensional diameters in no indicator lesion increase by ⩾25%. A classification of PR was also made if the sum of the products of the largest perpendicular diameters of the indicator lesions decreased by ⩾50%, or if ⩾50% of nodular or plaque-like lesions became macules; if ⩾75% of predominantly nodular lesions flattened to indurated plaques or if criteria for a CR were met but lesion-associated oedema or effusion persisted. In addition, those patients with cutaneous CR in whom invasive restaging of visceral disease was contraindicated were considered to have had a PR.

Stable disease (SD) was defined as any response that did not meet criteria for CR, PR or disease progression.

Progressive disease (PD) was defined as the appearance of new lesions, an increase of ⩾25% in the size of previously existing lesions, a change in character from macular to plaque-like or nodular in ⩾25% of previously existing lesions or an increase in oedema or effusions.

Best response (BR) was defined as the highest level of response achieved by the patient at any time during treatment, using the decreasing scale CR, PR, SD and PD. Time to treatment failure was defined as the number of days between the beginning of treatment and the onset of progressive disease after the patient had achieved his best response.

Patients were evaluated weekly for toxicity; complete blood counts were performed within 48 h before dosing throughout the study. Biweekly efficacy evaluation included evaluation of tumour response (after at least two cycles of therapy), assessment of Karnofsky status and completion of the quality-of-life questionnaire. Patients were to be withdrawn from the study if KS did not respond or had progressed after four doses of SL-DOX. Patients were also to be withdrawn in the event of profound and persistent neutropenia, anaemia or thrombocytopenia, biochemical abnormalities, intercurrent illness interfering with participation, pregnancy, patient request, investigator discretion or evidence of cardiotoxity. Echocardiography was repeated after every four cycles when the cumulative dose of doxorubicin exceeded 400 mg $m^{-2}$.

Quality of life was evaluated in 214 patients using a 37-item self-assessment questionnaire that included five domains: functional ability (ten items), pain (two items), KS-specific questions (four items), body image (three items) and physical condition (18 items). The first seven questions were yes/no questions relating to activities of daily living. The remaining 30 items had various anchored categorical responses and addressed general physical health as well as issues related specifically to Kaposi's sarcoma and body image.

### *Regimen*

SL-DOX (Sequus Pharmaceuticals, Menlo Park, CA, USA) was administered intravenously in a 30 min infusion every 2 weeks at doses of 10 mg $m^{-2}$, 20 mg $m^{-2}$ or 40 mg $m^{-2}$. Patients began treatment at 10 mg $m^{-2}$ or 20 mg $m^{-2}$ depending on investigator discretion, and the dose was titrated upwards if KS lesions failed to respond. During the trial, dose could be titrated downward in the event of toxicity. As a result of disease progression, ten patients received doses of SL-DOX other than 10 mg $m^{-2}$, 20 mg $m^{-2}$ or 40 mg $m^{-2}$ at the discretion of the investigators.

Prophylaxis against *Pneumocystis carinii* pneumonia (PCP) was encouraged and concomitant nucleoside therapy permitted. Growth factor support was also permitted to manage neutropenia.

### *Statistical methods*

In the time-to-event analyses (cycle number at BR, time to BR, days to treatment failure, etc.) the mean, standard error, median and range are computed from Kaplan–Meier curves. In the analysis of the quality-of-life questionnaire, change within each patient from baseline to the time of the best KS response was analysed using a paired *t*-test.

### Results

### *Patients*

A total of 247 patients were enrolled. Patient demographics are given in Table I, baseline presentation of KS in Table II and TIS staging of KS in Table III. The median baseline CD4 count for the 228 patients in whom it was measured was 30.5 $mm^{-3}$. Of these patients, 199/228 (87.3%) had CD4 counts < 200 $mm^{-3}$.

One hundred and sixty-two patients (65.8%) received prophylaxis against opportunistic infection during the course of the trial. Of these 162 patients, 97 received sulphamethox-

**Table I** Patient demographics (*n* = 247)

| | |
|---|---|
| Age (mean ± s.e.) | 39.1 ± 0.56 |
| Male [*n* (%)] | 242 (98.0) |
| Race [*n* (%)] | |
| Caucasian | 230 (93.1) |
| Non-Caucasian | 17 (6.9) |
| AIDS risk factor | |
| Homosexuality | 210 (85.0) |
| IVDA[a] | 10 (4.0) |
| Other/Unknown | 27 (10.9) |
| $CD4^+$ $mm^{-3}$ (median)[b] | 30.5 |
| Karnofsky score, baseline (mean ± s.e.) | 75.8 ± 1.11 |

[a]IVDA, intravenous drug abuse. [b]CD4 counts were measured in 228/247 patients (92.3%).

**Table II** Presentation of Kaposi's sarcoma at baseline ($n=247$)

| *Presentation* | *Number of patients (%)*[a] |
|---|---|
| Skin/subcutaneous | 234 (94.7) |
| Oral lesions | 136 (55.1) |
| Visceral involvement | 95 (38.5) |
| Lung | 72 (29.1) |
| Gastrointestinal tract | 41 (16.6) |
| Lymph nodes | 32 (12.6) |
| Other/unknown | 17 (6.9) |

[a]Percentages sum to more than 100 since patients could have had lesions at more than one site.

**Table III** Tumour–immune system–systemic illness (TIS) staging of Kaposi's sarcoma at entry ($n=247$)

| *TIS stage* | n *(%)* |
|---|---|
| $T_0I_0S_0$ | 14 (5.7) |
| $T_0I_0S_1$ | 1 (0.4) |
| $T_0I_1S_0$ | 54 (21.9) |
| $T_1I_0S_0$ | 5 (2.0) |
| $T_0I_1S_1$ | 23 (9.3) |
| $T_1I_0S_1$ | 5 (2.0) |
| $T_1I_1S_0$ | 63 (25.5) |
| $T_1I_1S_1$ | 79 (32.0) |
| Unknown | 3 (1.2) |

azole/trimethoprim (59.9%), 82 (50.6%) pentamidine and 33 (20.4%) dapsone. One hundred and sixty-two patients were also treated with an anti-retroviral nucleoside either before or at the same time as SL-DOX therapy.

Of 247 patients enrolled, 167 (67.6%) received at least six cycles of SL-DOX. One hundred and two patients (41.3%) received at least 26 cycles of SL-DOX. The mean cumulative dose of SL-DOX administered was 144 mg $m^{-2}$ (median 120 mg $m^{-2}$; range 10–520 mg $m^{-2}$).

Best response for 238 patients for whom data are available is given in Table IV. One hundred and ninety-two patients (80.7%) achieved a complete or partial response (CI, 76% to 86%). Patients typically achieved their best response after less than three cycles of chemotherapy. The duration of PRs and/or CRs is illustrated in Figure 1. Treatment failure (defined as the development of progressive disease after the patient had achieved his best response) occurred in 73/238 patients or 30.7% (Table V). Patients whose BR was progressive disease were omitted from the time-to-treatment-failure analysis.

There was no statistically significant correlation between the percentage of patients who obtained a complete or partial response on the one hand and the baseline CD4 count ($<50$ $mm^{-3}$ *vs* $\geqslant 50$ $mm^{-3}$), baseline neutrophil count ($<2000$ $mm^{-3}$ *vs* $\geqslant 2000$ $mm^{-3}$) or baseline ACTG systemic disease status (good *vs* poor) (Krown *et al*., 1989) on the other.

*Quality-of-life*

Quality-of-life (QOL) questionnaires were available for 214 patients. Comparison of the mean total and mean domain scores at baseline to mean total and domain scores at the time patients achieved their best response showed statistically significant improvements in the following: total score ($P<0.001$), pain subscore ($P=0.02$), KS subscore ($P=0.02$) and physical condition subscore ($P=0.007$). No statistically significant changes in functional ability occurred.

*Patient outcomes*

Seventy patients (28.3%) died during the course of the trial. Eleven patient deaths were considered possibly related to the study drug by the investigators. In four of these 11 patients, the cause of death was listed as pneumonia, in two, pneumonia and sepsis, and in one patient each, generalised herpes simplex viral infection, cerebral bleeding, peritonitis

**Table IV** Best response to SL-DOX ($n=238$)

| | |
|---|---|
| Best response (BR) | |
| Complete | 15 (6.3) |
| Partial | 177 (74.4) |
| Stable | 44 (18.5) |
| Progression | 2 (0.8) |
| Cycle no. at BR | |
| Median | 1 |
| Mean (s.e.) | 2.3 (0.15) |
| Range | 1–20 |
| Time to BR (days) | |
| Median | 25.0 |
| Mean (s.e.) | 39.6 (2.9) |
| Range | 1–408 |

**Figure 1** Duration of response in patients with either PR or CR.

**Table V** Time to treatment failure ($n=238$)

| | |
|---|---|
| Number (%) of patients with treatment failure | 73 (30.7) |
| Days to treatment failure | |
| Median | 250 |
| Mean (s.e.) | 275.5 (14.8) |
| Range | 6*–504* |
| Cycle number at treatment failure | 1–26* |

*Censored observation (patient had not experienced treatment failure at indicated time point).

associated with a perforated duodenal ulcer and respiratory difficulty. The cause of death in one patient was listed as heart failure and in another as severe oesophagogastric candidiasis and drug-induced hepatitis.

*Adverse experiences*

Information on adverse experiences (AEs) is available for 245/247 patients (99.2%). A total of 1906 AEs were reported in 239 of the 245 patients. Ten patients (4.0%) withdrew from the study because of drug-related AEs or laboratory abnormalities.

Possible infusion reactions occurred in 13 of 245 patients and included dyspnoea, facial flushing, nausea and vomiting, chest pain, facial oedema and dizziness. In three of these patients, these reactions were judged by the investigators to be severe. These three patients, however, recovered from the reactions without sequelae. Two patients withdrew from the trial because of an infusion reaction. Non-infusion-related adverse experiences potentially related to SL-DOX are listed in Table VI.

Cardiovascular AEs judged probably or possibly related to SL-DOX therapy occurred in 21 of 245 patients (8.6%). These included four patients (1.6%) with hypotension, three (1.2%) with pericardial effusion, three (1.2%) with thrombophlebitis and two each (0.8%) with heart failure and tachycardia. No patients were withdrawn from the study because of cardiovascular abnormalities although as noted above, one patient died of cardiac failure considered possibly related to study drug by the investigator. Four patients (1.6%) developed palmar–plantar erythrodysaesthesia during the course of therapy.

*Clinical laboratory abnormalities*

Clinical laboratory data were available for 247 patients (100%). Grade 3 or 4 neutropenia ($<1000\ mm^{-3}$) occurred during 281 of 2023 cycles of chemotherapy (13.9%). Grade 3 or 4 thrombocytopenia ($<50\,000\ mm^{-3}$) occurred during 29 cycles (1.4%). Incidence and degree of myelotoxicity are summarised in Table VII. Four patients were withdrawn from the study because of neutropenia.

Treatment with SL-DOX caused increases in alkaline phosphatase (AP), aspartate aminotransferase (AST) and total bilirubin levels. The mean ± s.d. baseline alkaline AP level for the 245/247 patients for whom data were available was $172.0 \pm 136$ IU $L^{-1}$. At maximal individual AP levels, the mean value rose to $327.0 \pm 364.5$ IU $L^{-1}$ for the 236 patients for whom data were available. In terms of AST, the mean ± s.d. baseline value for 228 patients for whom data were available was $25.1 \pm 27.8$ IU $L^{-1}$, and at maximal individual AST values, the mean value rose to $44.3 \pm 49.1$ IU $L^{-1}$ for the 232 patients for whom data were available. The mean ± s.d. bilirubin level at baseline was $0.5 \pm 0.4$ mg $dl^{-1}$ (for 237 patients) and rose at maximal individual values to a mean ± s.d. of $0.9 \pm 1.2$ mg $dl^{-1}$ (for 238 patients). One patient in the study died of acute hepatitis thought to be possibly related to SL-DOX. However, no patient was withdrawn from the study because of liver function test abnormalities.

**Table VI** Adverse experiences during SL-DOX therapy ($n = 245$)

| Adverse event | Number (%) of patients: All AEs | Number (%) of patients: Probably/possibly related to SL-DOX |
|---|---|---|
| Nausea | 62 (25.3) | 49 (20.0) |
| Vomiting | 34 (13.9) | 21 (8.6) |
| Stomatitis | 29 (11.8) | 23 (9.4) |
| Constipation | 20 (8.2) | 7 (2.9) |
| Diarrhoea | 61 (24.9) | 28 (11.4) |
| Alopecia | 29 (11.8) | 28 (11.4) |

**Table VII** Incidence and degree of post-baseline neutropenia, thrombocytopenia and anaemia by dose group

| | n (%) |
|---|---|
| Total patients | 247 |
| Minimum neutrophil count($10^3\ mm^{-3}$) | |
| ≥1 | 103 (42.9) |
| 0.5 to <1.0 | 94 (39.2) |
| <0.5 | 43 (17.9) |
| Not available | 7 |
| Mean (s.e.) | 1.0 (0.04) |
| Minimum platelet count ($10^3\ mm^{-3}$) | |
| ≥50 | 212 (88.0) |
| 25 to <50 | 13 (5.4) |
| <25 | 16 (6.6) |
| Not available | 6 |
| Mean (s.e.) | 140.9 (5.10) |
| Minimum haemoglobin (g $dl^{-1}$) | |
| ≥8 | 189 (78.4) |
| 6.5 to <8 | 41 (17.0) |
| <6.5 | 11 (4.6) |
| Not avaliable | 6 |
| Mean (s.e.) | 9.5 (0.12) |

The minimum value obtained during the course of the trial was selected for each measurement. Means and s.e. were computed from patients' minima. Percentages are based on number of patients for whom data were available.

*Opportunistic infection*

A total of 286 individual events categorised as opportunistic infections were reported in 131 of 245 patients (53.5%). Oral, oesophageal and disseminated candidiasis represented 115 of the 286 events. There were 44 events reported of cytomegaloviral (CMV) infection during the study, including CMV retinitis (22 events), gastrointestinal CMV (13 events), pulmonary CMV (3 events) and 6 unspecified events of CMV. In addition, there were 57 events of herpetic infection, 24 events of PCP pneumonia, 23 events of mycobacterial infection, 12 events of toxoplasmosis and 10 events of systemic fungal infection other than candidiasis.

### Discussion

The use of conventional chemotherapy in the treatment of epidemic KS has been limited in large measure by toxicity. For example, doxorubicin- and etoposide-containing regimens can be strongly myelosuppressive and agents that are relatively marrow-sparing, such as vincristine, vinblastine and bleomycin, may be burdened by other important toxicities such as peripheral neuropathy or pulmonary fibrosis. When myelosuppressive regimens are used, treatment with antiviral drugs such as zidovudine and ganciclovir may have to be suspended.

Liposomal doxorubicin has demonstrated efficacy against a variety of murine tumours including human xenografts (Vaage *et al.*, 1992). Phase I clinical trials in cancer patients showed that Stealth liposomal doxorubicin had a long plasma half-life and increased accumulation in malignant effusions relative to comparable doses of conventional doxorubicin (Rahman *et al.*, 1990; Gabizon *et al.*, 1994). These findings suggested that Stealth liposomal doxorubicin could have significant advantages over the conventional formulation of doxorubicin in the treatment of KS patients.

In this study, over 80% of patients had a best response of either CR or PR when SL-DOX was administered biweekly. These results compare favourably with those obtained in a study involving 53 AIDS patients with KS given 15 mg $m^{-2}$ conventional doxorubicin weekly (Fischl *et al.*, 1993).

Only two patients completed this study from which 47 of 53 evaluable patients were withdrawn after a median treatment duration of 2.7 months. Seventeen of these patients (32%) were withdrawn because of toxicity and 13 (24.5%) were withdrawn because of progressive KS. Among 50 patients evaluable for efficacy, none had a CR, five (10%) had a PR, 32 (64%) had a minor response, 12 (24%) no change and one (2%) progression as their best response.

In a second study involving 33 patients who were treated biweekly with a regimen incorporating conventional doxorubicin (10 mg $m^{-2}$ or 20 mg $m^{-2}$), bleomycin 10 mg $m^{-2}$ and vincristine (1.4 mg $m^{-2}$, 2 mg maximum) (Gill *et al.*, 1990*b*), 26/33 patients (79%) had either a partial (18 patients) or complete (eight patients) response. These responses were achieved after a median of six cycles. In terms of toxicity, 11 patients (33%) developed neutrophil counts $<1000\ mm^{-3}$, 19 (58%) experienced nausea and/or vomiting and 20 (61%) developed alopecia. Vincristine-related neuropathy occurred in 20 patients (61%) and in two the severity of the neuropathy required discontinuation of vincristine after one or two cycles.

Other, non-anthracycline-containing chemotherapeutic regimens have employed etoposide, vincristine, vinblastine or bleomycin as single agents (Laubenstein *et al.*, 1984; Mintzer *et al.*, 1985; Volberding *et al.*, 1985; Lassoued *et al.*, 1990), or two-drug combinations of vincristine and bleomycin (Gill *et al.*, 1990*a*), or vincristine and vinblastine (Kaplan *et al.*, 1986). Alpha-interferon is also used as a single agent

(Gelmann et al., 1987; Lane et al., 1988). Although it is difficult to compare results across clinical trials (in part because of differences in the criteria used to assess response and the small size of many of these trials), these regimens generally appear to be less effective than combination therapy incorporating doxorubicin.

In this trial, there was no statistically significant correlation between the percentage of patients who achieved PR or CR and the baseline CD4 count, neutrophil count or ACTG systemic illness status. However, the majority of patients in this trial were severely immunocompromised (as indicated by the large percentage with a poor ACTG immune system status at baseline), and it is conceivable that a significant difference might have been observed had the study included a larger number of less severely ill patients.

The activity and acceptable toxicity of SL-DOX in the treatment of AIDS-KS suggest potential advantages of liposomal drug delivery in this malignancy. In a pilot study of liposomal daunorubicin (Presant et al., 1993), 2 of 24 evaluable patients (8.3%) had a complete response and 13 (54.2%) had a partial response. Nine patients (37.5%) had either no response or progressive disease. Myelossuppression was the most common adverse event in that trial, with 11 of 25 patients evaluable for toxicity (44.0%) experiencing grade 3 or 4 granulocytopenia.

Myelosuppression is also the most important toxicity of SL-DOX. In this study, 137 of 240 patients (57.1%) for whom data were available experienced either grade 3 or 4 neutropenia during at least one cycle of therapy. Only a minority of cycles (13.9%) were complicated by grade 3 or 4 neutropenia, however, and only two cases of drug-related septic infection were reported.

The elevations in hepatic enzyme levels that were observed in this study were not clearly related to SL-DOX. It is possible, however, that in certain cases elevation of serum alkaline phosphatase was related to HIV-induced cholestasis (Payne et al., 1991) aggravated in some way by the clearance of the doxorubicin-containing liposomes by the reticuloendothelial system of the liver.

The patient in whom fulminant hepatic necrosis was ascribed to SL-DOX has been described elsewhere (Hengge et al., 1993). This patient, who entered the study with elevated levels of hepatic enzymes, suffered from chronic active hepatitis B infection and had been heavily treated with fluconazole for oesophageal candidiasis shortly before his death. It was postulated that the hepatic necrosis seen in this patient resulted from the combined effects of drug- and virus-induced hepatitis.

Palmar–plantar erythrodysaesthesia syndrome was described in four patients in this trial. This syndrome is typically manifest as a prodrome of dysaethesias involving the hands and feet in which an initial tingling progresses over 3–4 days to discomfort and then to pain which is accompanied by symmetrical swelling and erythema of the palms, fingers and soles. If therapy is interrupted, resolution takes place over 5–14 days with desquamation of the affected skin. This syndrome has been reported to occur both with SL-DOX (Gordon et al., 1995) and with continuous infusion of doxorubicin (Lokich and Moore, 1984). In one series of 36 patients who received long-term, low-dose continuous infusion of doxorubicin (Vogelzang and Ratain, 1985), 15 of 32 patients treated for more than 30 days developed erythrodysaesthesia. This suggests a causal relationship between the persistence of doxorubicin in the blood and the occurrence of the syndrome. Such a relationship is consistent with the significantly prolonged $t_{1/2}$ and AUC for doxorubicin that are achieved by incorporating this agent in Stealth liposomes (Northfelt et al., 1995).

Since the mean cumulative anthracycline exposure in this trial was relatively low (144 mg $m^{-2}$), significant cardiotoxicity would not have been expected. Moreover, the occurrence of HIV-associated cardiomyopathy (Grody et al., 1990) complicates interpretation of the adverse cardiac experiences observed here.

The 53.5% incidence of opportunistic infection on therapy in this trial compares favorably with rates in most trials of other chemotherapeutic regimens. In these trials, opportunistic infections occurred in 11–89% of patients (Laubenstein et al., 1984; Gill et al., 1990a,b; Fischl et al., 1993; Gill et al., 1991). Moreover, given that 80.6% of all patients had CD4 counts on entry < 200 $mm^{-3}$ and that at least a third of all trial participants received no form of prophylaxis against *Pneumocystis carinii* pneumonia, the 24 cases of PCP reported here would not have been unanticipated (Masur et al., 1989; Fischl et al., 1988; Hirschel et al., 1991; Freedberg et al., 1991). The same holds true for the 44 cases of cytomegalovirus-related disease given the relationship between the probability of developing such disease and CD4 counts < 100 $mm^{-3}$ (Gallant et al., 1992).

A trial comparing single-agent therapy with SL-DOX to combination therapy with doxorubicin, vincristine and bleomycin (ABV) is ongoing and will assess the relative safety and efficacy of these two regimens. Phase I trials of SL-DOX have indicated that SL-DOX has activity in breast, ovarian, head and neck, prostatic, renal cell and non-small-cell lung carcinomas (Uziely et al., 1995).

SL-DOX provides meaningful clinical benefits in the single-agent therapy of AIDS-KS. At a dose of approximately 20 mg $m^{-2}$ in biweekly cycles, it provides the ability to induce partial or complete responses in a high percentage of patients, responses that are achieved with manageable toxicity and which, in certain cases, can be prolonged. Even brief responses, however, can be meaningful, given the degree of discomfort or disability experienced by these patients and their frequently short life expectancies. This is suggested by the statistically significant effects on quality of life at the best KS response. It is likely that the ability specifically to deliver relatively high doses of doxorubicin to tumour tissue with SL-DOX will have important implications in the treatment of other malignancies.

**Acknowledgements**
This study has been supported by Sequus Pharmaceuticals, Menlo Park, CA, USA.

**Appendix**

*The International SL-DOX Study Group*

Australia: D A Cooper and S Milliken, St Vincent's Hospital, Sydney; D Goldstein, Prince Henry Hospital, Sydney.

United Kingdom: BK Mandal, North Manchester General Hospital, Manchester; MF Spittle, The Middlesex Hospital, London; JSW Stewart, St Mary's Hospital, London.

Germany: W Brockhaus, Klinikum der Stadt Nuremberg, Nuremberg; J Th Fischer, Klinikum Karlsruhe, Karlsruhe; J Bogner, F-D Goebel, Medizinische Poliklinik der Universität Munich, Munich; M Goos, M Baumann, Universitätsklinikum Essen, Essen; H Jablonowski, H Szelenji, Medizinische Einrichtungen der Heinrich Heine Universität, Düsseldorf; P Kern, Medizinische Universitätsklinik und Poliklinik, Ulm; H Knechten, Praxiszentrum, Aachen; M L'Age, Auguste-Viktoria-Krankenhaus, Berlin; WN Meigel, Allgemeines Krankenhaus St Georg, Hamburg; PS Mitrou, Johann-Wolfgang-Goethe-Universität, Frankfurt; T Gabrysiak, I Schedel, Klinikum der Medizinische Hochschule Hannover, Hannover; N Spannbrucker, Universitätsklinik Bonn, Bonn; HJ Stellbrink, Universität Krankenhaus Eppendorf, Hamburg.

Italy: F Milazzo, G Rizzardini, Ospedale Luigi Sacco, Milan.

The Netherlands: JCC Borleffs, Academic Hospital Utrecht, Utrecht.

Portugal: F Antunes, L Caldeira, Hospital de Sante Maria, Lisbon.

Switzerland: M Opravil, Universitätsspital Zurich, Zurich.

### References

CENTERS FOR DISEASE CONTROL UPDATE. (1986). Acquired immunodeficiency syndrome (AIDS) – United States. *MMWR*, **35,** 757 – 760, 765 – 766.

DES JARLAIS DC, STONEBURNER R, THOMAS P AND FRIEDMAN SR. (1987). Declines in proportion of Kaposi's sarcoma among cases of AIDS in multiple risk groups in New York City (letter). *Lancet*, **2,** 1024 – 1025.

FISCHL MA, DICKINSON GM AND LA VOIE L. (1988). Safety and efficacy of sulfamethoxazole and trimethoprim chemoprophylaxis for *Pneumocystis carinii* pneumonia in AIDS. *JAMA*, **259,** 1185 – 1189.

FISCHL MA, KROWN, SE, O'BOYLE KP, MITSUYASU R, MILES S, WERNZ JC, VOLBERDING PA, KAHN J, GROOPMAN JE, FEINBERG J, WOODY M AND THE AIDS CLINICAL TRIALS GROUP. (1993). Weekly doxorubicin in the treatment of patients with AIDS-related Kaposi's sarcoma. *J. Acquir. Immune Defic. Syndr.*, **6,** 259 – 264.

FREEDBERG KA, TOSTESON AN, COHEN CJ AND COTTON DJ. (1991). Primary prophylaxis for Pneumocystis carinii pneumonia in HIV-infected people with CD4 counts below 200/mm$^3$: a cost-effectiveness analysis. *J. Acquir. Immune Defic. Syndr.*, **4,** 521 – 531.

GABIZON A, CATANE R, UZIELY B, KAUFMAN B, TAMAR S, COHEN R, MARTIN F, HUANG A AND BARENHOLZ Y. (1994). Prolonged circulation time and enhanced accumulation in malignant exudates of doxorubicin encapsulated in polyethylene-glycol coated liposomes. *Cancer Res.*, **54,** 987 – 992.

GALLANT JE, MOORE RD, RICHMAN DD, KERULY J, CHAISSON RE AND THE ZIDOVUDINE EPIDEMIOLOGY STUDY GROUP. (1992). Incidence and natural history of cytomegalovirus disease in patients with advanced human immunodeficiency virus disease treated with zidovudine. *J. Infect. Dis.*, **166,** 1223 – 1227.

GELMANN EP, LONGO D, LANE HC, FAUCI AS, MASUR H, WESLEY M, PREBLE OT, JACOB J AND STEIS R. (1987). Combination chemotherapy of disseminated Kaposi's sarcoma in patients with the acquired immune deficiency syndrome. *Am. J. Med.*, **82,** 456 – 462.

GILL PS, RARICK MU, BERNSTEIN-SINGER M, HARB M, ESPINA BM, SHAW V AND LEVINE A. (1990*a*). Treatment of advanced Kaposi's sarcoma using a combination of bleomycin and vincristine. *Am. J. Clin. Oncol. (CCT)*, **13,** 315 – 319.

GILL PS, RARICK MU, ESPINA B, LOUEIRO C, BERNSTEIN-SINGER M, AKIL B AND LEVINE AM. (1990*b*). Advanced acquired immune deficiency syndrome-related Kaposi's sarcoma. *Cancer*, **65,** 1074 – 1078.

GILL PS, RARICK M, MCCUTCHAN JA, SLATER L, PARKER B, MUCHMORE E, BERNSTEIN-SINGER M, AKIL B, ESPINA BM, KRAILO M AND LEVINE A. (1991). Systemic treatment of AIDS-related Kaposi's sarcoma: results of a randomized trial. *Am. J. Med.*, **90,** 427 – 433.

GORDON KB, TAJUDDIN A, GUITART J, KUZEL TM, ERAMO LR AND VONROEN J. (1995). Hand foot syndrome associated with liposome-encapsulated doxorubicin therapy. *Cancer*, **75,** 2169 – 2173.

GRODY WW, CHENG L AND LEWIS W. (1990). Infection of the heart by the human immunodeficiency virus. *Am. J. Cardiol.*, **66,** 203 – 206.

HENGGE UR, BROCKMEYER HN, RAßHOFER R AND GOOS M. (1993). Fatal hepatic failure with liposomal doxorubicin (letter). *Lancet*, **341,** 383 – 384.

HIRSCHEL B, LAZZARIN A, CHOPARD P, OPRAVIL M, FURRER HJ, RUTTIMANN F, VERNAZZA P, CHAVE JP, ANCARANI F AND GABRIEL V. (1991). A controlled study of inhaled pentamidine for primary prevention of *Pneumocystis carinii* pneumonia. *N. Engl. J. Med.*, **324,** 1079 – 1083.

KAPLAN L, ABRAMS D AND VOLBERDING P. (1986). Treatment of Kaposi's sarcoma in acquired immunodeficiency syndrome with an alternating vincristine – vinblastine regimen. *Cancer Treat. Rep.*, **70,** 1121 – 1122.

KROWN SE, METROKA C AND WERNZ JC. (1989). Kaposi's sarcoma in the acquired immune deficiency syndrome: a proposal for uniform evaluation, response, and staging criteria. *J. Clin. Oncol.*, **7,** 1201 – 1207.

LANE HC, KOVACS JA, FEINBERG J, HERPIN B, DAVEY V, WALKER R, DEYTON L, METCALF JA, BASELER M AND SALZMAN N. (1988). Anti-retroviral effects of interferon-alpha in AIDS-associated Kaposi's sarcoma. *Lancet*, **2,** 1218 – 1222.

LASSOUED K, CLAUVEL J.-P, KATLAMA C, JANIER M, PICARD C AND MATHERON S. (1990). Treatment of the acquired immune deficiency syndrome-related Kaposi's sarcoma with bleomycin as a single agent. *Cancer*, **66,** 1869 – 1872.

LAUBENSTEIN LJ, KRIGEL RL, ODAJNYK CM, HYMES KB, FRIEDMAN-KIEN A, WERNZ JC AND MUGGIA FM. (1984). Treatment of epidemic Kaposi's sarcoma with etoposide or a combination of doxorubicin, bleomycin, and vinblastine. *J. Clin. Oncol.*, **2,** 1115 – 1120.

LOKICH JJ AND MOORE C. (1984). Chemotherapy-associated palmar – plantar erythrodysesthesia syndrome. *Ann. Int. Med.*, **101,** 798 – 800.

MASUR H, OGNIBENE FP, YARCHOAN R, SHELHAMER JH, BAIRD BF, TRAVIS W, SUFFREDINI AF, DEYTON L, KOVACS JA, FALLOON J, DAVEY R, POLIS M, METCALF J, BASELER M, WESLEY R, GILL VJ, FAUCI AS AND LANE HC. (1989). CD4 counts as predictors of opportunistic pneumonias in human immunodeficiency virus (HIV) infections. *Ann. Int. Med.*, **111,** 223 – 231.

MINTZER DM, REAL FX, JOVINO L AND KROWN SE. (1985). Treatment of Kaposi's sarcoma and thrombocytopenia with vincristine in patients with the acquired immunodeficiency syndrome. *Ann. Int. Med.*, **102,** 200 – 202.

NORTHFELT DW, KAPLAN LD, RUSSELL J, VOLBERDING PA AND MARTIN FJ. (1995). Pharmacokinetics and tumor localization of DOX-SL$^{TM}$ (Stealth® liposomal doxorubicin) by comparison with Adriamycin in patients with AIDS and Kaposi's sarcoma. In *Stealth®* Liposomes, Lasic D and Martin F. (eds) pp. 257 – 266. CRC Press: Boca Raton.

PAPAHADJOPOULOS D, ALLEN TM, GABIZON A, MAYHEW E, MATTHAY K, HUANG SK, LEE K.-D, WOODLE MC, LASIC DD, REDEMANN C AND MARTIN FJ. (1991). Sterically stabilized liposomes: improvements in pharmacokinetics and antitumor therapeutic efficacy. *Proc. Natl Acad. Sci. USA*, **88,** 11460 – 11464.

PAYNE TH, COHN DL, DAVIDSON AJ, HENRY TD, SCHAEFFER JW AND GABOW PA. (1991). Marked elevations of serum alkaline phosphatase in patients with AIDS. *J. Acquir. Immune Defic. Syndro.*, **4,** 238 – 243.

PETERS BS, BECK EJ, COLEMAN DG, WADSWORTH MJH, MCGUINNESS O, HARRIS JRW AND PINCHING AJ. (1991). Changing disease patterns in patients with AIDS in a referral centre in the United Kingdom: the changing face of AIDS. *Br. Med. J.*, **302,** 203 – 207.

PRESANT CA, SCOLARO M, KENNEDY P, BLAYNEY DW, FLANAGAN B, LISAK J AND PRESANT J. (1993). Liposomal daunorubicin treatment of HIV-associated Kaposi's sarcoma. *Lancet*, **341,** 1242 – 1243.

RAHMAN A, TREAT J, ROH J.-K, POTKUL LA, ALVORD WG, FORST D AND WOOLLEY PV. (1990). A phase I clinical trial and pharmacokinetic evaluation of liposome-encapsulated doxorubicin. *J. Clin. Oncol.*, **8,** 1093 – 1100.

UZIELY B, JEFFERS S, ISACSON R, KUTSCH K, WEI-TSAO D, YEHOSHUA Z, LIBSON E, MUGGIA FM AND GABIZON A. (1995). Liposomal doxorubicin (DOX-SL®): antitumor activity and unique toxicities during two complementary phase I studies. *J. Clin. Oncol.*, **13,** 1777 – 1785.

VAAGE J, MAYHEW E, LASIC D AND MARTIN F. (1992). Therapy of primary and metastatic mouse mammary carcinomas with doxorubicin encapsulated in long circulating liposomes. *Int. J. Cancer*, **51,** 942 – 948.

VOGELZANG NJ AND RATAIN MJ. (1985). Cancer chemotherapy and skin changes (letter). *Ann. Int. Med.*, **103,** 303 – 304.

VOLBERDING PA, ABRAMS DI, CONANT M, KASLOW K, VRANIZAN K AND ZIEGLER J. (1985). Vinblastine therapy for Kaposi's sarcoma in the acquired immunodeficiency syndrome. *Ann. Int. Med.*, **103,** 335 – 338.

VOLBERDING PA, KUSICK P AND FEIGAL DW. (1989). Effect of chemotherapy for HIV associated Kaposi's sarcoma on long-term survival (abstract no. 11). In *Program and abstracts of the 8th Annual Meeting of the American Society of Clinical Oncology.* Chicago, IL: American Society of Clinical Oncology, pp. 3.